# Shear Bond Strength of Three Composite Resins to Fluorosed and Sound Dentine: In Vitro Study

**DOI:** 10.1155/2020/4568568

**Published:** 2020-04-09

**Authors:** Sabra Jaâfoura, Amira Kikly, Saida Sahtout, Mounir Trabelsi, Dorra Kammoun

**Affiliations:** ^1^Department of Dental Biomaterials, ABCDF Laboratory, Faculty of Dental Medicine, University of Monastir, Avicenne Avenue, Monastir 5000, Tunisia; ^2^Department of Conservative Odontology, Laboratory of Dento-Facial, Clinical and Biological Approach (ABCDF) LR12ES10, Faculty of Dental Medicine, University of Monastir, Avicenne Avenue, Monastir 5000, Tunisia

## Abstract

**Introduction:**

This in vitro study compared the shear strength of three composite resin systems to fluorosed and normal dentin.

**Methods:**

Silorane FiltekTM P90, FiltekTMZ250 XT in combination with the adhesive system AdperTM Single bond 2, and Amelogen® Plus in association with Peak Universal Bond® were tested. Fifteen normal and 15 fluorosed dentine disks were prepared per material. The shear bond strength test was performed using a universal machine.

**Results:**

One-way ANOVA revealed significant differences in bond strength between the tested composite resins. All tested materials had significantly different adhesion at the fluorosed and the nonfluorosed interface. FiltekTM Z250 XT and Silorane had lower adhesion values to fluorosed than to normal dentin. In contrast, Amelogen® Plus presented a better average resistance at the fluorosed interface.

**Conclusion:**

Amelogen® Plus presented a better average shear bond strength on the fluorosed dentine. FiltekTMZ250 XT showed the best adhesion forces and shear bond strength with sound dentine. Further studies are needed to better understand the sealing of these systems.

## 1. Introduction

Dental fluorosis is a specific disturbance due to chronic ingestion of excessive fluoride during the formative period of the dentition [1]. The use of fluoride in preventive dentistry has been the most effective anticaries measure, but it is also associated with the increasing prevalence of dental fluorosis in many countries. Excessive fluoride ingestion during enamel maturation adversely affects cleavage and removal of enamel proteins, such as amelogenins [2]. Retention of proteins and water interferes with enamel crystal growth, resulting in varying degrees of subsurface porosities [3]. In fluorotic dentine of permanent teeth, there is increased interglobular dentine formation and accentuation of incremental lines of von Ebner [4]. Distinct changes in mineralization pattern are not confirmed in the fluorotic enamel. Conversely, the underlying fluorotic dentine exhibits accentuated incremental growth patterns. After cessation of enamel secretion, a substantial variation of mineral content can be observed in dentine with occasional bands of inter globular dentine [5, 6]. Fluoride has been shown to alter the adsorption of proteoglycans and glycosaminoglycans or the noncollagenous proteins to hydroxyapatite. This may affect the inhibition of the growth of crystals in various directions that decides the shape [7].

The current adhesive systems obtain acceptable micromechanical retention between resin and dentine in two different ways. The first method utilizes acid etching for demineralization of subsurface intact dentine and complete removal of the smear layer. The second method, called the self-etch approach, integrates usage of monomers that are slightly acidic. This leads to partial demineralization of the smear layer and the underlying dentin, hence incorporating the demineralized remnants of the smear layer to be used as a bonding substrate. There has been a growing trend to move toward simplified, consolidated bonding systems from the original type of multicomponent systems over the last few years [8]. It was reported that microtensile bond strength decreased with the severity of fluorosis [9]. Shear bond strength is an interesting test for adhesion [10]. Thus, information on bond strength of resin composite to fluorosed dentine gains interest. Up to now, only limited and contradictory data are available on shear bond strength to fluorosed human dentine. Further research is needed to clarify these conflicting results.

Therefore, the aim of this in vitro study was to evaluate the effects of fluorosis on shear bond strengths of three composite resins and their corresponding adhesives. Our null hypothesis was that fluorosis does not affect shear bond strengths of dental adhesives bonded to dentin.

## 2. Materials and Methods

### 2.1. Adhesive-Composite Resin Systems Used in This Study

The adhesive-composite resin systems used in this study are listed in Table 1. Silorane FiltekTM P90's adhesive (3M ESPE, St. Paul, USA) is a two-step self-etching adhesive system. FiltekTM Z250XT was used with AdperTM Single Bond 2 (3M ESPE, St. Paul, USA) which is a two-step acid-etch system. Amelogen® Plus was used with Peak SE Primer combined with Peak® Universal Bond (Ultradent, Inc, South Jordan, USA) constituting a two-step self-etching adhesive system. All the bonding systems were used according to the manufacturer's instructions.

### 2.2. Method

Forty-five sound and forty-five fluorosed human molars extracted from different subjects between the ages of 30 and 40 were used for this study. The teeth were caries-free and had been extracted due to periodontal, orthodontic, or prosthetic reasons. Dental fluorosis severity was assessed according to the Thylstrup-Fejerskov Index (TFI) [11]. Only teeth with TFI 1-3 were selected (Thylstrup).

Before extraction, informed consent to use the teeth for the study was obtained from the subjects. Teeth were preserved in saline immediately after extraction. Then, the teeth were cleaned of tissue debris and scale deposits and stored again in physiological saline. Each tooth was embedded in a self-curing resin cube then cut in the mesio-distal direction, following the coronal-apical axis at a parapulpaire plane. Only one section was performed on each tooth.

**Figure 1:**
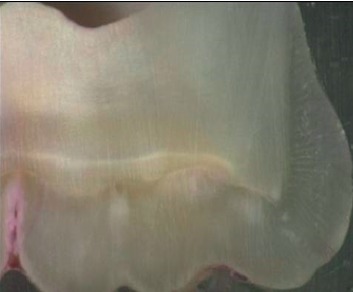
Delimitation of the surface provided for bonding and verification of the presence of a 2.4 mm diameter dentine area.

Teeth were sectioned by means of a slow rotating diamond bur (Isomet, Buehler, Lake Bluff, IL, USA) set at a cutting speed of 7 rev/min under irrigation. To carry out the bonding step, the bonding surface was defined at the proximal area under a stereomicroscope (Figure 1).

The sections were randomly assigned to different groups. The following distribution was adopted:

- 15 sound surfaces and 15 fluorosed surfaces received resin discs of FiltekTMSilorane P90
- 15 sound surfaces and 15 fluorosed surfaces received resin discs of FiltekTMZ250 XT
- 15 sound surfaces and 15 fluorosed surfaces received resin discs of Amelogen® Plus

For each type of resin, the bonding was done on purely dentine surfaces.

In order to place the composite resin onto the tooth surface, hollow silicone molds of 2.4 mm in diameter and 4 mm in height were used. Each mold was placed on a tooth surface and then filled with the corresponding composite resin following the bonding procedure recommended by the manufacturer for each composite resin system. Two resin inputs were performed to maximize the depth of cure throughout the thickness of the material. The light-curing unit characteristics are detailed in Table 2.

For FiltekTM Silorane P90, the bonding protocol was as follows:

- Apply the primer and massage the surface for 15 seconds
- Light curing for 10 seconds
- Application of bonding
- Light curing for 10 seconds
- Placement of the mold on the surface provided for bonding
- Mold filling (two intakes) and light-curing

For FiltekTMZ250 XT, the following bonding procedure was applied:

- Etch the dental surface with 37% orthophosphoric acid for 15 seconds
- Rinse for 15 seconds
- Pat dry with a cotton ball for 10 seconds
- Apply two layers of adhesive and spread with an air jet for 10 seconds
- Light cure for 10 seconds
- Place the hollow mold on the surface covered with bonding
- Apply Filtek™Z250 XT, shade A3; the thickness of the layer is 2 mm and the duration of light-curing for 20 seconds

**Figure 2:**
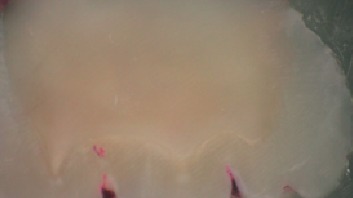
Slightly moistened bonding surface.

As for Amelogen® Plus, we proceeded as follows:

- Activate the Peak® SE primer syringe by forcibly pushing the dated piston into the central cylinder
- Moisten the bonding surface (Figure 2)
- Apply the Peak® SE primer with the mini black brush tip for 20 seconds on the bonding surface with a continuous scouring movement on the dentine
- Dry for 3 seconds using an air/water syringe
- Apply a thick layer of Peak® universal adhesive with the Inspiral® brush tip, shaking gently for 10 seconds
- Dry for 10 seconds using an air jet, the surface should take on a shiny appearance
- Light cure for 10 seconds
- Place Amelogen® Plus in successive layers of approximately 2 mm in thickness and light-cure each layer for 20 seconds *s* until the mold is filled

**Figure 3:**
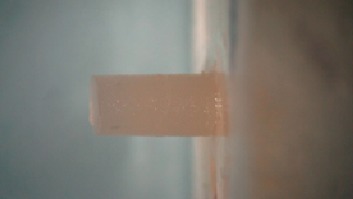
Specimen ready for shear strength test.

The sample obtained after bonding (dental section surmounted by the resin disk (Figure 3) subsequently underwent a shear strength test (shear bond strength (SBS)) at the tooth material interface to determine the type of fracture and calculate the shear strength at the breaking load.

Generally, fracture mode is classified into three types—type 1: adhesive failure between adhesive resin and dentine; type 2: partially adhesive failure between adhesive resin and dentine, including cohesive failure in the adhesive resin; and type 3: cohesive failure in the resin composite.

The shear strength was measured using the universal testing machine (H5KS Model HTN-5000N, England) equipped at its upper plate with a sharp blade. The sample was fixed on the lower plate of the device. The lowering speed was 1 mm/min.

Shear strength is defined as the ratio of the load incurring shear failure (F in Newton) and the area of the disc material at the interface (in mm^2^).

Statistical analysis was performed using a data processing software for Windows: SPSS 17.0. Two statistical tests were used: one-way ANOVA and Tukey test. The average difference was considered significant at the 0.05 level.

## 3. Results and Discussion

### 3.1. Results

The descriptive statistics on the shear bond strength (MPa) of the composite resin systems are presented in Table 3. Analysis of variance (ANOVA) indicated a significant difference between the different materials (*p* < 0.05) (Table 4).

The highest values of shear bond strengths were measured in FiltekTM Z250 in sound dentine, and in Amelogen®Plus in fluorosed dentine. Multiple comparisons showed that the shear bond strength in Silorane was significantly lower than in Amelogen® Plus (*p* < 0.05) for fluorosed dentine. No significant difference was found between Amelogen® Plus and Silorane in sound dentine (*p* > 0.05) (see Table 5).

Adhesion forces to the surfaces of fluorosed teeth followed this descending order: Amelogen® Plus, FiltekTM Z250, and Silorane. In sound dentine, the order was as follows: FiltekTM Z250, Amelogen® Plus, and Silorane.

When comparing fluorosed and sound dentine per adhesive system, the SBS values for FiltekTM Silorane P90 system were slightly higher on nonfluorosed dentine. The variation was significant (*p*=0.05).

The resistance of the bonded joint to fluorosed dentine, with the Z250 system, was significantly lower (*p* < 0.000) compared to nonfluorosed dentine. Amelogen® Plus showed significantly higher SBS values when bonded to teeth with fluorosis (*p* < 0.000).

**Figure 4:**
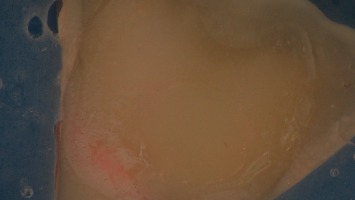
Observation of the bonding surface after rupture.

The observation of the bonding interface after rupture showed for all samples an adhesive failure: the composite was detached from the dentine surface with its adhesive. The cohesive force of the composite material was greater than the force of adhesion to the tooth structure (Figure 4).

### 3.2. Discussion

This study was designed to evaluate the effects of fluorosis on shear bond strengths of composite resins in comparison with sound dentine.

Since fluoride content can vary between different teeth [12], only fluorosed human molar teeth were used in this study.

Three composite resin systems were chosen for this study. They belong to the same class of microhybrid composite resin [13]. Two were based on methacrylate and one based on Silorane. Silorane-based composites were developed with the intention of solving the problems of polymerization shrinkage and water absorption. FiltekTM Z250 is a material that has proven good adhesion to dental structures in comparison with other systems. This material in combination with AdperTM Single Bond 2 is used as a control in several studies [14].

All the adhesives used contain ethanol in their compositions, thus requiring active application to penetrate the adhesive. Pleffken et al. (2011) suggest that there are significant differences depending both on the type of adhesive and its method of application. All the adhesive systems studied showed significant differences. The active application of two layers of self-etching bonding systems gives better results than the passive application method [15].

Researchers are inclined to use shear bond and microtensile methods as well as fracture mechanics to understand the properties of the adhesive interfaces of dentine. To assess the quality of dental adhesives performance on the bond, bond strength tests are necessary [10, 16].

Compared to normal teeth, fluorosed teeth have more fluoride and less calcium. High concentrations of fluoride reduced the mineralization rate of the teeth. The dentine fluoride level was positively correlated with the size of the dentinal tubuli, which affects the mechanical locking of an adhesive to the dentinal surface [12]. A substantial variation of mineral content can be observed in dentine with occasional bands of interglobular dentine. Waidyasekera et al. (2007) found that the existing dentine bonding systems offer lower bond strengths to mildly and moderately fluorosed dentine tissue [9].

In our study, the shear strength values obtained with 37% phosphoric acid for 15 seconds showed a significant difference between the fluorosed and nonfluorosed teeth. This result is consistent with the findings of Adanir et al. (2009) [17]. The shear strength obtained with Silorane and Filtek Z250 on the fluorosed dentine was significantly lower than on the nonfluorosed dentin. This significant difference is consistent with the result of Ermis et al. (2003) [18]. This can be attributed to fluoridated apatite, which is less soluble in acid.

Mechanical anchorage is offered by etching with phosphoric acid in the case of AdperTM Single Bond 2. The packaging of the dentine with orthophosphoric acid offers more adhesive strength in the case of a resin-based composite resin material than the acid monomers [19]. Polyacrylic acid in AdperTM Single Bond 2 promotes chelation with calcium and the formation of hydrogen bridges with dentine components. This may be a significant factor leading to higher shear bond strength values. Another component possibly responsible for the high bond strength values is the 5 nm silica nanofiller incorporated at 10% weight in AdperTM Single Bond 2 adhesive. These particles may have a role in the formation of a resin film that stabilizes the hybrid layer [20].

For the other 2-step self-etching adhesive, the acid potential of the hydrophilic monomers, such as the methacrylic esters of phosphoric acid, is low (pH from 0.8 to 2.5) and allows only surface demineralization. Thus, the acid monomers of the self-etching adhesives have a lower attack potential than the mineral acid at the same concentration and, therefore, a less pronounced mechanical anchoring [21]. Self-etching adhesive systems combine primer and bonding. The primer is dried with the air. This act leads to the solubilisation of calcium and phosphate ions that are, hence, suspended in alcoholic solvents and water from the primer. After these volatile solvents are evaporated, the concentration of calcium and phosphate may be greater than the solubility constant of the calcium phosphate product, resulting in its precipitation within the primer. This limits the ability of the adhesive to penetrate the surface treated by the primer, resulting in lower bond strength values [22]. In our study, etching with 37% orthophosphoric acid further weakened the dentine surface, in comparison with etching by Peak® SE Primer. This may account for the superiority of the shear strength of Amelogen® Plus.

There were comparisons carried out on the microtensile bond strength of resin composite to teeth with either mild, moderate, or severe fluorosis [23]. It was observed that the two-step self-etching adhesive (Clearfil SE Bond/Kuraray Medical) generally had higher bond strength than the all-in-one bonding system (Clearfil Tri S Bond/Kuraray Medical) and the etch-and-rinse bonding system (Single Bond/3M-ESPE).

The hydrophobic coating of the Silorane adhesive can provide additional stability at the bond interface, reducing the amount of water absorption through time. But the hydrophilic monomer (HEMA) tends to group before polymerization to create a hydrophilic zone [24, 25]. Water can rapidly dilute the adhesive and affect the structure of the polymer thereby forming hydrophilic polymers which allow a movement of the water molecules from the dentine to the adhesive layer. Knowing that the fluorosis dentine exhibits a nonhomogeneous mineral distribution with zones of hypocalcification, the dentinal tubuli arrange the mineral elements of the dentine, thus favouring a better absorption of water. This phenomenon plasticizes the polymer and reduces the mechanical properties, resulting in a significant reduction of linkage forces. The low removal capacity of the smear layer is related to the high pH value (2.7), which is insufficient to etch the intact dentine [26]. This may be the reason for low values observed for Silorane [27].

For all samples, the fracture was adhesive. According to Kimmes et al. (2010), Adper Single Bond 2 shows an adhesive-type fracture in 90% of cases, and Peak SE shows an adhesive-type fracture in 100% of cases [19]. Ermis and Gokay (2003) investigated the effect of dental fluorosis on shear bond strength of a composite material to dentin. An adhesive mode of failure was most prevalent in fluorosis-free teeth. They concluded that fluorosis does not affect the shear bond strength of composite material to human dentine [18].

It must be emphasized that this study was performed in vitro. Therefore, shear bond strengths obtained in this study may not very well coincide with clinical successes. Further in vivo and clinical studies are still needed to substantiate the results obtained in this study.

## 4. Conclusions

This in vitro study concluded that Amelogen® Plus presented a better average shear bond strength on the fluorosed dentine. FiltekTMZ250 XT showed the best adhesion forces and shear bond strength with sound dentine. Further studies are needed to better understand the sealing of these systems.

## Figures and Tables

**Table 1:** Composition of different composite resins used and their adhesive system.

Composite resin	Adhesive system
Silorane FILTEKTM P90	Primer (pH = 2.7)—2-hydroxyethyl methacrylate (HEMA), bisphenol-A diglycidyl ether dimethacrylate (BIS-GMA), water, ethanol, phosphoric acid–methacryloxy-hexylesters mixture, silane-treated silica, 1,6-hexanediol dimethacrylate, copolymer of acrylic and itaconic acid, (dimethylamino) ethyle methacrylate, dl-camphoroquinone
3,4-Epoxy-cyclohexylethyl-cyclo-polymethylsiloxane; bis-3 ,4-epoxy-cyclohexylethyl-phenylmethylsilane; silanised quartz; yttrium fluoride; camphoroquinone	Bonding: substituted dimethacrylate, silane-treated silica, triethylene glycol dimethacrylate (TEGDMA), phosphoric acide methacryloxy-hexylesters, dl-camphoroquinone, 1,6-hexanediol dimethacrylate

FiltekTM Z250 XT	orthophosphoric acid: etchant: phosphoric acid 35 %, water, silica
Bis-GMA; UDMA (urethanedimethacrylate); bis-EMA (ethyl-methacrylate bisphenol-A), silica, zirconia (60% in weight)	AdperTM Single Bond 2: HEMA, water, ethanol, amines, bis-GMA, methacrylate-functional, polycarboxylic acid, dimethacrylates. silanated colloidal, silica (10% in weight)

Amelogen® Plus	Peak® SE primer: 2-hydroxyethyl methacrylate, ethylic alcohol
Bis-GMA, TEGDMA, barium, boron, aluminium (0.4–0.7 *μ*m)	Peak® Universal Bond: deshydrated alcohol, 2-hydroxyethyl methacrylate, methacrylic acide, chlorhexidine

**Table 2:** Characteristics of the light-curing unit used.

Light-curing unit	Manufacturer	Wave length	Power
ST-10B®	Ultradent	420 à 480 nm	>1000 mW/cm^2^

**Table 3:** Descriptive statistics of shear bond strength.

		*N*	Mean value	Standard deviation	Minimum	Maximum
SBS nonfluorosed dentine	Silorane	15	6.8289	3.17399	1.55	13.15
	Z250	15	22.4395	6.80485	13.99	33.49
	Amelogen	15	7.3477	5.01340	1.33	19.34
	Total	45	12.2053	8.91859	1.33	33.49
SBS fluorosed dentine	Silorane	15	4.9400	1.61899	2.00	8.50
	Z250	15	10.2400	3.73283	4.90	19.70
	Amelogen	15	15.6533	5.01439	8.00	29.50
	Total	45	10.2778	5.72998	2.00	29.50

**Table 4:** One-way anova of the shear bond strength at the dentine interface.

		Sum of square	Ddl	Mean sum of square	*F*	*p* value
SBS nonfluorosed dentine	Intergroup^*∗*^	2358,612	2	1179,306	43,402	0.000
	Intragroup^*∗∗*^	1141,200	42	27,171		
	Total	3499,812	44			
SBS fluorosed dentine	Intergroup^*∗*^	860,848	2	430,424	30,966	0.000
	Intragroup^*∗∗*^	583,789	42	13,900		
	Total	1444,638	44			

^*∗*^The intergroup variance stands for variance between group means and the overall mean (group: composite resin system). ^*∗∗*^The intragroup variance stands for variance between group means and each group data.

**Table 5:** Multiple comparisons of the different systems tested.

	(*I*) Material	(*j*) Material	*p* value	95% confidence interval	
				Lower bound	Superior bound
SBS nonfluorosed dentine	Silorane	Z250	0.000	−20.2349	−10.9863
		Amelogen	0.960	−5.1431	4.1055
	Z250	Silorane	0.000	10.9863	20.2349
		Amelogen	0.000	10.4675	19.7161
	Amelogen	Silorane	0.960	−4.1055	5.1431
		Z250	0.000	−19.7161	−10.4675
SBS fluorosed dentine	Silorane	Z250	0.001	−8.6074	−1.9926
		Amelogen	0.000	−14.0207	−7.4059
	Z250	Silorane	0.001	1.9926	8.6074
		Amelogen	0.001	−8.7207	−2.1059
	Amelogen	Silorane	0.000	7.4059	14.0207
		Z250	0.001	2.1059	8.7207

## Data Availability

The data used to support the findings of this study are included within the article.
